# The association between tryptophan levels and postpartum mood disorders: a systematic review and meta-analysis

**DOI:** 10.1186/s12888-022-04178-6

**Published:** 2022-08-08

**Authors:** Zhao Feng Liu, Amy Sylivris, Michael Gordon, Suresh Sundram

**Affiliations:** 1 Department of Psychiatry, School of Clinical Sciences, Monash University, Melbourne, VIC Australia; 2 Mental Health Program, Monash Health, Melbourne, VIC Australia; 3 Monash Medical Centre, Block P, Level 3, 246 Clayton Rd, Melbourne, 3168 VIC Australia

**Keywords:** Postpartum Depression, Postpartum Mood Disorder, Tryptophan Level, Tryptophan metabolism

## Abstract

Over 50% of women experience mood disturbance in the postpartum period, with significant implications for maternal and infant health but identifying those at risk is not easily possible. The essential amino acid, tryptophan (TRP) through its neuroactive metabolites, has been implicated in the pathology of mood disorders. Thus, TRP levels tested in the peripartum period have been proposed as a potential biomarker for subsequent development of postpartum mood disturbances, in particular postpartum depression (PPD). A systematic review and meta-analysis following PROSPERO guidelines [CRD42021252462] was conducted on peer-reviewed, English language studies that measured blood levels of TRP during the postpartum period in women who were also evaluated for postpartum “blues” or PPD. Thirteen studies met the inclusion criteria, of which five studies contained sufficient data to conduct a meta-analysis. Low total TRP levels in postpartum days 1 to 5 were significantly associated with PPD (SMD: -5.39, 95%CI [-7.72, -3.05]). No significant association was found between free TRP levels in the postpartum period and PPD (SMD: -3.43, 95%CI [-7.76, 0.89]). Our findings confirm the necessity for more replicable designed studies regarding TRP and its relationship to postpartum depression. If there were greater clarity regarding TRP metabolism during pregnancy, then the next step would be to consider measuring total plasma TRP levels on postpartum days 1 to 5 to identify women at greater risk of developing PPD.

**Supplementary Information:**

The online version contains supplementary material available at 10.1186/s12888-022-04178-6.

## Introduction

When Major Depressive Disorder (MDD) occurs in the postpartum period it has been historically considered a subtype of MDD. Although it is diagnosed using the same symptom profile [1], its onset, usually within 6 months after childbirth [2], and prevalence affecting up to 13% of women [3], suggest that postpartum depression (PPD) may have specific aetiological factors distinct from MDD.

Evolving and variable definitions of postpartum depression (PPD) have been presented regarding its timing of onset in the Diagnostic and Statistical Manual of Mental Disorder and International Classification of Diseases [1, 4, 5]. Many existing studies define PPD with onset exclusively in the postpartum period, with timeframes ranging up to one year after delivery [2]. However, epidemiological evidence suggests that the increased risk of unipolar depression following delivery extends up to 6 months [6–8]. Consistent with these data but acknowledging definitional heterogeneity, we defined PPD for this systematic review as the onset of a major depressive episode in the first 6 months postpartum. Moreover we sought to examine changes in TRP metabolism in the postpartum period and its association with PPD, thus limiting our range to changes exclusively postpartum, consistent with most extant studies on the relationship between TRP and PPD [9].

Postpartum depression may have significant ramifications for the health of the mother, development of the child and the family unit. For the mother, PPD is associated with poorer overall physical and psychological health [10], leading to decreased quality of life [11] and increased risk of addictive behaviour and suicidal ideation [12, 13]. For the child, untreated PPD is associated with impaired childhood development [14], manifesting with behavioural issues of excessive infant crying, temperamental difficulties and sleep problems [15], in addition to long-term impacts on cognitive functioning, behavioural inhibition and emotional maladjustment [16, 17]. At a societal level, mothers suffering from PPD required considerably more healthcare resources to care for them and their child [18]. The substantial personal, family, social, and economic impacts of PPD justify a need to better characterise and address the aetiological factors and in particular, identify putative biomarkers that can assist in early identification of this debilitating condition.

Postpartum “blues” is conceptualised as a more common and less intense manifestation of PPD. It is characterized by transient and mild dysphoria peaking during the first week postpartum [19]. There are currently no universally-established criteria for the diagnosis of postpartum blues, thus estimates of its prevalence range from 13.7 to 76.0% depending on the criteria employed [20]. Although postpartum blues is considered a relatively benign condition that does not warrant treatment [21], it is of clinical relevance because it is predictive of the subsequent development of PPD [22].

The high prevalence, implications, and timing of PPD have prompted a plethora of studies investigating its aetiology with hopes of guiding prevention and development of new treatments in the future. One proposed aetiological mechanism involves alterations in the metabolism of the essential amino acid, tryptophan (TRP), over the peripartum period [23]. This is because of its role as the precursor to serotonin and other neuroactive agents [24] and its involvement in a variety of psychiatric conditions including Major Depressive Disorder [25, 26]. Tryptophan is metabolised through four pathways: the kynurenine, hydroxylation, decarboxylation and transamination pathways [27–29]. The kynurenine pathway accounts for 95% of dietary TRP metabolism; of which 90% occurs in the liver via the hepatic enzyme TRP 2,3-dioxygenase (TDO), and to a much lesser extent elsewhere via the extrahepatic enzyme indoleamine 2,3-dioxygenase (IDO) [30].

Plasma TRP levels can be measured as either free or total, with the majority of plasma TRP existing in an albumin-bound state. Total plasma TRP refers to the combination of free and albumin-bound fractions [31]. Typically, concentrations of total and free TRP are closely related due to the rapid equilibration between the two fractions. Amino acid transporters facilitate the movement of tryptophan and other amino acids competitively across the blood brain barrier. Hence, TRP availability to the brain is most readily expressed by the tryptophan to competing amino acid (CAA) ratio, further subdivided into [total TRP]/[CAA] and [free TRP]/[CAA] [32].

Tryptophan disposition and metabolism throughout pregnancy is influenced by four main factors: foetal growth; the placental role in immune tolerance; altered partitioning in the blood; and hormonal fluctuations.

Firstly, during pregnancy, TRP is important for the production of protein, and for foetal development [30]. Previous studies have reported that in healthy women without PPD, total TRP gradually declines during the antenatal period, reaching a trough immediately before delivery. After parturition, total TRP rises rapidly and peaks on days 1 and 2 postpartum, followed by a slower return to normal [33, 34]. This may be partially driven by increased foetal demand in late pregnancy [35, 36].

Alternatively, a recent 2021 review collated evidence regarding the role of the placenta in tryptophan metabolism [37]. The decline in TRP may be a physiological result of the placenta, where it has been shown that localisation of IDO occurs [38]. TRP catabolism via the IDO and TDO pathways are important for establishing and maintaining foetal-maternal immune tolerance during pregnancy [39]. Consequently, the depletion of TRP by the placenta may predispose the mother to a range of psychiatric and neurological disorders [40].

Furthermore, TRP availability to the brain is affected by binding to albumin. In mid to late pregnancy, physiological depletion of albumin and elevation of non-esterified fatty acids (NEFA) cause displacement of bound TRP and increased tissue uptake, thus causing a decrease in total TRP levels [31].

Finally, pregnancy alters hormone and enzyme levels in the body. In particular, an increase in cortisol levels during the late pregnancy and postpartum period has been shown to decrease total TRP levels [23, 33]. Cortisol induces the activity of TDO, which as mentioned, accounts for nearly 95% of overall TRP degradation [30]. Furthermore following high levels of progesterone and oestrogen during pregnancy, there is a physiological drop in these hormones during the postpartum period, which may have a two-fold influence on mood disorders. First, due to the antidepressant properties of progesterone and oestrogen [41], lower postpartum levels have been linked to an increased risk of development of postpartum blues [42]. Additionally, progesterone and oestrogen inhibit TDO [43], therefore pregnancy is considered a period of long-term TDO suppression. It has been posited that post-partum there is a rebound corticosteroid-induced activation of TDO resulting in increased TRP degradation during the early postpartum period [44].

Pregnancy results in increased metabolic demand for TRP, altered albumin and NEFA levels, and hormonal fluctuations; all of which potentially contribute to the steady decline of total serum TRP levels throughout pregnancy [35, 36]. A deficit in total TRP holds significant implications for the development of depressive symptoms. Tryptophan is the amino acid precursor of 5-hydroxytryptamine (5-HT) in the lesser, but clinically significant serotonin pathway [45, 46]. Low total TRP means that we can predict circulating levels of 5-HT and its downstream metabolites to be likewise decreased [47]. This is consistent with deficiencies of 5-HT being implicated in major depression [46, 48].

To date, there has not been any meta-analysis comparing total and free TRP levels post-delivery. This meta-analysis aims to determine the relationship between TRP and PPD and postpartum blues.

## Methods

### Registration and study protocol

 This study was conducted with reference to the Preferred Reporting Items for Systematic Reviews and Meta-analysis (PRISMA) checklist. The protocol was registered through PROSPERO (CRD42021252462).

### Search strategy

The literature search comprised 2 stages. First, Cochrane Library, Scopus, Web of Science, Embase, OVID Medline and PsycINFO were systematically searched from inception until 17 October 2021. A combination of Medical Subject Heading (MeSH) terms and/or text keywords were used and adapted to each database. Further details of the complete search strategy can be found in Additional file 1: Appendix A. In addition, manual checking of reference lists in all eligible studies for any further relevant studies was undertaken.

### Study selection

After all records were organised and duplicates excluded, selection of eligible studies was conducted by two reviewers (AS and ZL) independently. Inclusion criteria were: (1) reported in a peer-reviewed journal, (2) involved a cohort of postpartum women diagnosed with PPD or postpartum blues using screening questionnaires and/or diagnostic assessments, (3) reported a blood test for TRP levels during the postpartum period, and (4) the full-text was published in English. Exclusion criteria were: (1) study populations with a pre-existing diagnosis of depression or a diagnosis made in the antenatal period, (2) studies published in a language other than English, (3) antenatal complications, and (4) overlapping samples.

The study selection process occurred in 2 stages: first, title and abstract screening of all records; then, full text articles were retrieved and screened for inclusion from this list of potential eligible records. During title and abstract screening, if the reviewers were unable to determine whether the article met the inclusion and exclusion criteria, then the article was retrieved for full text review. Disagreements in selection criteria during the full text review stage were resolved by discussion between the two reviewers (AS and ZL) and, if necessary, a third reviewer (SS).

### Data collection

 Data were extracted by two reviewers (AS and ZL) independently using a standardised excel template. This included study characteristics (study design, year of publication, sample size, diagnostic criteria used and endpoints), patient characteristics for potential subgroup analyses (number of participants, age, mode of delivery, marital status, socioeconomic status, pre-existing health conditions, medications), and relevant outcome measurements (timing of the postpartum blood test, type of TRP (total or plasma), TRP level, regression values). All measures of TRP were expressed using the SI unit of mol/l and standard error of the mean was converted to standard deviation.

We contacted authors for additional data if the study itself appeared eligible for inclusion, but the necessary data were unpublished. If authors did not respond or no data was available, then the study was excluded from the meta-analysis.

To analyse as much of the existing literature as possible, we conducted an additional descriptive analysis of all studies deemed eligible after full-text screening. This aimed to include the studies which were identified as eligible but did not provide sufficient data for inclusion in the meta-analysis. Two reviewers (AS and ZL) independently summarised the key findings of the eligible papers after full-text screening. The information extracted for qualitative assessment included: (1) the study and patient characteristics (as above), and (2) the key conclusions of the study.

### Quality assessment

For quality assessment in this study, the Newcastle-Ottawa Scale (NOS) [49] was used which is one of the more common tools for non-randomised controlled trials [50]. However, there are no quality assessment tools which adequately cover a case-series design, so for the purposes of our assessment we treated case-series studies as single-arm cohort studies. From the NOS, four stars or less were considered “poor” quality, five or six stars were considered “fair”, and studies attaining seven or more stars were classified as “good” quality.

### Statistical analysis

The studies were assessed in two parts, for correlation coefficients relating depression scores with tryptophan levels, and separately for continuous data comparing depressed and control post-partum mothers.

For data obtained from correlation coefficients, the correlation coefficients themselves served as the effect size. The Hedges-Olkin method with Fisher z transformation of the Pearson correlation coefficient was utilised [51]. The variance of Fisher’s Z is given by the reciprocal of the sample size minus three.

For continuous measures, mean, standard deviation and numbers of participants were compared with control data for the meta-analysis as described in Borenstein et al. 2009 [52]. Unstandardised effect sizes were calculated using Hedge’s g with random effects model, with DerSimonian–Laird method and Knapp–Hartung standard error adjustment. Prediction intervals were also calculated.

Statistical analysis was performed using STATA, version 17.0 [53].

## Results

### Study selection and study characteristics

**Fig. 1:**
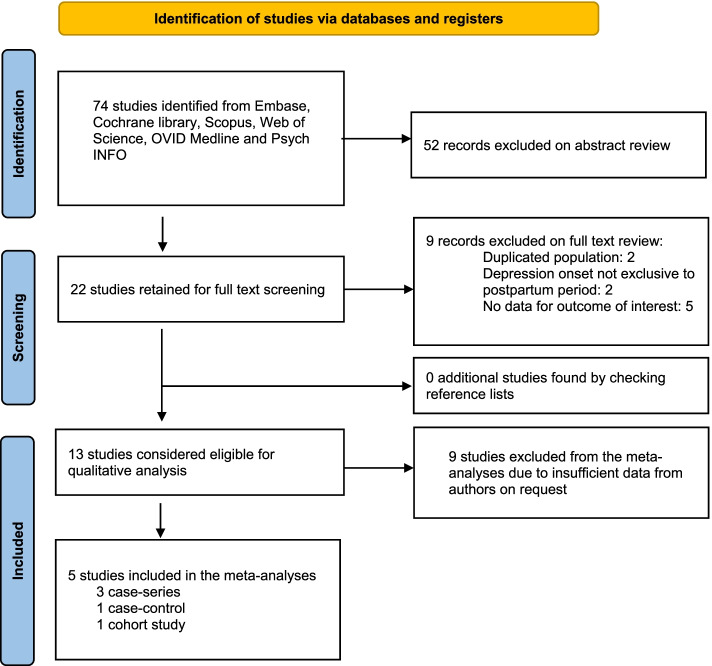
Search strategy used to identify studies eligible for inclusion in the present systematic review and meta-analysis

We initially identified 74 studies (Fig. 1) from the available databases, thirteen studies of which were considered eligible for inclusion in the descriptive analysis. Ten reported data on the association between plasma TRP levels and PPD [23, 33, 34, 54–60], and six studies evaluated the association between plasma TRP levels and postpartum blues [33, 34, 57, 61–64].

However, of these thirteen eligible studies identified from the literature search, only five supplied sufficient data for inclusion in the meta-analyses. These studies included comparative data for TRP levels post-delivery and the development of PPD [34, 54, 57, 58, 60].

All included studies are summarised in Table 1.

Table 2 summaries the analyses performed.

**Table 1:** Summary of the eligible studies, including study and population characteristics and outcome variable extracted

	Design	Population	TRP* levels	Diagnostic Tool	Data Extracted	Qualitative Results
Stein 1976 [54]	Cohort	16 non-pregnant women 18 women with postpartum blues	Day 6: Total Free	Day 1–7: Total Affective Score^a^	Total TRP: PPD* Free TRP: PPD	Significant negative correlation for free TRP and PPD, but no significant results for total TRP and PPD
Handley 1977 [61]	Case-series	18 postpartum women	Day 3–5: Free	Day 3–5: MAACL*, BDI*	N/A	Significant negative correlation between free TRP and depression score in immediate postpartum period (days 1–5)
Handley 1980 [33]	Case-series	71 postpartum women	Days 1–5 and week 6: Total Free	Days 1–5 and week 6: BDI, MAACL, VAS*	N/A	PPD cohort: - Significant positive correlation between incidence of depression at 6 months and a failure to show a rise in total TRP within 2 days of parturition. - No statistically significant findings for free TRP Blues cohort: - Blues associated with failure to show early total TRP peak on days 1–2 - Significant negative association between free TRP and blues, with extremely low seasonal free TRP levels from January to April
Gard 1986 [34]	Case-series	14 women with postpartum blues vs. 28 women who were symptom-free 11 women with PPD vs. 26 women who were symptom-free	Days 1–5: Total Free	Days 1–5: MAACL, BDI, Clinical interview, Self-reporting	Free TRP: PPD	Significant negative correlation between postpartum blues and decreased total TRP in immediate postpartum (day 1–5 after delivery) Blues cases showed smaller rise in total TRP on day 1 postpartum No significant correlation between free TRP and PPD
Maes 1992 [23]	Cohort	31 postpartum women 19 non-pregnant women	Day 3: Total	Day 3: ZDI*, BDI, STAI*	N/A	Significant negative correlation between total TRP and depression scores Significant negative correlation between TRP/CAA and depression scores
Abou-Saleh 1999 [55]	Cohort	38 non-pregnant women 23 pregnant women 62 postpartum women	Day 7: Free	Day 7: EPDS*	N/A	Significant negative correlation between free TRP and EPDS score, both measured on day 7 in postpartum women Postpartum women had significantly lower free TRP levels than non-pregnant controls
Maes 2001 [56]	Cohort	13 non-pregnant women 98 pregnant women	Days 1–3: Total	Days 1–3: ZDI, STAI	N/A	No significant relationship between total TRP and depression scores No significant relationship between TRP/CAA and depression scores
Kohl 2005 [57]	Case-series	95 postpartum women	Days 2 and 4: Total Free	Days 2 and 4: EPDS	Total TRP: PPD	Significant positive correlation between high EPDS score and a failure to show a rise in total TRP within 2 days of parturition
Bailara 2006 [62]	Case-series	50 postpartum women	Day 3: Unspecified	Day 3: Kennerly and Gath	N/A	Significant correlation between TRP/CAA and postpartum blues in immediate postpartum period (defined as day 1–5 after delivery)
Doornbos 2008 [64]	Case-series	26 postpartum women	Day 5 and week 6: Unspecified	Day 5: Kennerly and Gath	N/A	No significant difference in TRP/CAA between cases and non-cases of postpartum blues
Scrandis 2008 [58]	Case-series	27 postpartum women	Days 1–5 and weeks 5–6: Total	Days 1–5 and weeks 5–6: SIGH-SAD*	Total TRP: PPD	Significant correlation between total TRP and depression score
Veen 2016 [59]	Case-control	23 women with PPD 29 matched controls	Day 22–95: Unspecified	Within 3 months: EPDS	N/A	No difference in TRP levels between controls and cases with PPD
Duan 2019 [60]	Case-control	48 women with PPD 48 postpartum women without PPD	Day 1 and 3: Free	Week 8: EPDS	Free TRP: PPD	No significant difference in free TRP levels between PPD-cases and controls, on day 1 On day 3, free TRP levels in the PPD group was significantly higher

*PPD postpartum depression, *TRP tryptophan, *CAA competing amino acids, *ZDS Zung Depression Score, *VAS visual analogue scale, *MAACL Multiple Affect Adjective Checklist, *BDI Beck Depression Inventory, *EPDS Edinburgh Postnatal Depression Score, *STAI State-Trait Anxiety Inventory, *SIGH-SAD Structured Interview Guide for the Hamilton Depression Rating Scale-Seasonal Affective Disorder

^a^ Total Affective Score: cumulative score consisting of five symptoms (tearfulness, depression, anxiety, appetite, insomnia) ranked on a Likert scale

**Table 2:** Studies included within each analysis

Meta-analysis	
Total TRP and PPD	Scrandis 2008 [58] Kohl 2005 [57] Stein 1976 [54]
Free TRP and PPD	Duan 2018 [60] Gard 1986 [34] Stein 1976 [54]
**Descriptive analysis**	
Total TRP and PPD	Scrandis 2008 [58] Kohl 2005 [57] Maes 2001 [56] Maes 1992 [23] Stein 1976 [54] Handley 1980 [33]
Free TRP and PPD	Duan 2018 [60] Veen 2016 [59] Abou-Saleh 1999 [55] Gard 1986 [34] Handley 1980 [33] Stein 1976 [54]
Total TRP and postpartum blues	Kohl 2005 [57] Gard 1986 [34] Handley 1980 [33]
Free TRP and postpartum blues	Gard 1986 [34] Handley 1980 [33] Handley 1977 [61]
CAA/TRP and postpartum blues	Doornbos 2008 [64] Bailara 2006 [62]

### Quality assessment

All studies included in the systematic review were classified as either “fair” (*n* = 8) or “good” (*n* = 5) according to the Newcastle Ottawa Scale (Table 3). Of the eight “fair” studies, one [54] used an unvalidated tool to determine depression scores; whilst the other seven were “case series”, in which the control group was also exposed to the intervention (pregnancy). Quality assessment of these studies were performed under the category of cohort studies, but due to the study design they have an inherent inability to control for confounders between the groups.

**Table 3:** Quality assessment of eligible studies using NOS star system

	Selection (/4)	Comparability (/2)	Outcome (/3)	Total (/9)	Assessment
Stein 1976 [54]	★★★	★	★★	6	Fair
Handley 1977 [61]	★★		★★★	5	Fair
Handley 1980 [33]	★★		★★★	5	Fair
Gard 1986 [34]	★★★		★★★	6	Fair
Maes 1992 [23]	★★★	★	★★★	7	Good
Abou-Saleh 1999 [55]	★★★★	★	★★	7	Good
Maes 2001 [56]	★★★★	★	★★★	8	Good
Kohl 2005 [57]	★★★		★★★	6	Fair
Bailara 2006 [62]	★★		★★★	5	Fair
Doornbos 2008 [64]	★★★		★★★	6	Fair
Scrandis 2008 [58]	★★		★★★	5	Fair
Veen 2016 [59]	★★★	★	★★★	7	Good
Duan 2018 [60]	★★★	★★	★★★	8	Good

### Results: meta-analyses

A total of six studies provided descriptive data on the association between total plasma TRP and PPD [23, 33, 54, 56–58], but only 3 provided sufficient quantitative data to be included in the meta-analysis [54, 57, 58].

**Fig. 2:**
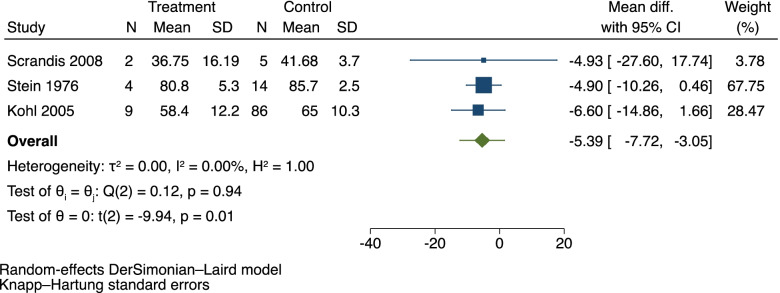
Total tryptophan levels in PPD vs. control post-partum controls. Prediction Interval marked in green whiskers on summary diamond

We conducted a meta-analysis comparing total TRP levels in mothers with PPD and control subjects (Fig. 2). We found a significant decrease in the total TRP levels during the immediate postpartum period (days 1 to 5 after birth) for women who developed PPD as compared to women who did not, with a mean difference of -5.39 µmol/L [95% CI -7.72, -3.05]. There was a low level of heterogeneity with I^2^ = 0% in the analysis. The 95% prediction interval for the mean difference was between − 12.27 and 1.50.

**Fig. 3:**
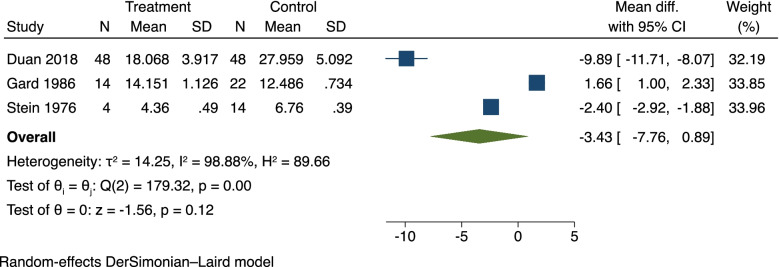
Random-effects meta-analysis of free Tryptophan levels comparing immediate postpartum TRP and PPD. The prediction interval is shown as whiskers on the mean summary diamond

We conducted a second meta-analysis on three studies which provided data comparing depressed mothers and controls where free plasma TRP was measured immediately post-partum [34, 54, 65]. There was no significant difference (SMD: -3.43, 95%CI [-7.76, 0.89]) in free TRP levels between the depressed and non-depressed mothers (Fig. 3).

We identified six studies which reported correlation coefficients related to TRP levels and continuous depression scores [33, 54, 55, 58, 61, 62]. Two studies reported a Spearman’s rank coefficient [33, 61], two studies reported Pearson correlation coefficients [55, 62] and the other two did not specify the type of correlation co-efficient. As a correlation meta-analysis requires Pearson’s correlations and TRP measures (brain TRP availability index, free TRP, total TRP or unspecified in two studies) varied, a correlation meta-analysis could not be conducted. Instead, the descriptive outcomes are presented below.

### Descriptive analysis

#### Postpartum depression and total TRP

Four of the six correlation studies found a significant negative correlation between total plasma TRP levels and depression scores in the postpartum period [23, 33, 57, 58], whereas two out of six failed to find any significant association [54, 56].

Three studies measured depression scores within 5 days of delivery with two studies published by the same group with different patient cohorts [23, 56]. Their first study (*n* = 31) found a significant negative correlation between total plasma TRP levels and the Zung Depression Score, whereas their larger second study (*n* = 98) did not. Both studies employed comparable methodologies including diurnal timing of blood sample collection and plasma assays. Notably the earlier study excluded two patients with very high Zung Depression Scores (ZDS > 40) to avoid perceived skewing of their results. Similar to the Maes et al. [23] study, the Scrandis et al. (2008) study [58] (*n* = 27), also found a significant negative correlation between total plasma TRP and the Hamilton Depression Rating Scale. However, the population under evaluation in this study only included high-risk women with either a positive history of recurrent mood or anxiety disorders (but no formal diagnosis of MDD) or a stressful social situation considered high risk for PPD. On the other hand, both earlier studies [23, 56] excluded women with any current or prior psychiatric history.

In contrast, an early, small study [54] (*n* = 18) found no significant correlation between total plasma TRP and depression scores measured on days 7 and 8 but did find a significant negative correlation between free plasma TRP and depression scores. The study, however, did not use a validated depression scale, but instead developed its own symptoms questionnaire called the “Total Affective Score” restricting its comparability to other studies.

Given the normal surge in TRP levels in the immediate postpartum period, two early studies observed an absence of this initial spike in PPD cases [33, 34]. One study (*n* = 63) sorted subjects into two groups, one where the initial TRP spike was clearly present, and the other where it was not. It found that the latter group had a significantly higher prevalence of PPD within the first 5 days postpartum (Chi^2^ = 18.37, *P* < 0.001), and up to 6 months postpartum (Chi^2^ = 5.6, *P* < 0.02) [33]. A more recent study (*n* = 95) found that total plasma TRP levels increased rapidly within 2 days postpartum in the control group, whereas no increase was seen in the group with high EPDS, the difference between the two groups reached statistical significance (*P* = 0.027) [57].

#### Postpartum depression and free TRP

Six studies provided descriptive data on the association between free plasma TRP and PPD [33, 34, 54, 55, 59, 60], of which three studies provided sufficient data to be included in the meta-analysis [34, 54, 60].

Of four studies examining the relationship between free plasma TRP and depression scores in the early postpartum period within 10 days of delivery, three found a significant negative correlation [54, 55, 60] and one study found no significant association [34]. Of the three studies with significant findings, one study [54] as noted above, employed its own unvalidated depression symptom questionnaire but the other two utilised the Edinburgh Postnatal Depression Scale (EPDS). The first of these (*n* = 23) applied an EPDS cut-off score of 12 on day 7 postpartum [55], and the second study (*n* = 96) applied a cut-off score of 13 [60]. This latter study found a significant negative correlation on day 3 postpartum but failed to do so on day 1.

Two studies measured the incidence of depressive episodes in the late postpartum period, at 2 and 6 months respectively. The first study [33] (*n* = 63) discussed in Postpartum depression and total TRP section, found a significant association between low total plasma TRP on days 1 and 2 postpartum and presentations of depression up to 6 months postpartum but failed to find the same association with free plasma TRP. The second study [59] (*n* = 46) found that cases of PPD, diagnosed through EPDS screening then clinical interviews, had lower levels of free plasma TRP on day 63 postpartum, but failed to reach significance. The study also confirmed the finding that postpartum women had significantly lower levels of plasma TRP compared to non-pregnant controls.

#### Postpartum blues and total TRP

Three studies provided descriptive data on the association between total plasma TRP and postpartum blues [33, 34, 57].

All three studies found postpartum blues to be significantly correlated with the absence of a physiological spike in total TRP levels on postpartum days 1 and 2. Two studies found the absence of the TRP spike to be significantly correlated with both postpartum blues and PPD, as previously described in Postpartum depression and total TRP section The first study (*n* = 71) utilised a combination of the Multiple Affect Adjective Checklist (MAACL), Beck Depression Inventory (BDI) and Visual Analogue Scale [33], whereas the second study (*n* = 95) screened for postpartum mood changes using the EPDS [57]. The third study (*n* = 52) also found total TRP levels to be significantly reduced in the blues group on day 1 postpartum [34], similarly using a combination of the MAACL, BDI and clinical interviews.

All three studies report consistent findings and support the hypothesis that the absence of an initial TRP spike is positively correlated with postpartum mood disturbance (previously described in Postpartum depression and total TRP section).

#### Postpartum blues and free TRP

Three studies provided descriptive data on the association between free plasma TRP and postpartum blues [33, 34, 61], two of which also reported on total plasma TRP as discussed in Postpartum blues and total TRP section.

Two studies were published by the same teams but with different patient cohorts, *n* = 18 [61] and *n* = 71 [33]. Both studies measured blues using a combination of MAACL, BDI, Visual Analogue Scale and clinical interviews. One British study [33] reported seasonal-specific results, with universally low levels of free plasma TRP found in women giving birth between January and April, irrespective of blues status. However, between May and December it found that women with postpartum blues had significantly lower levels of free plasma TRP compared to non-cases [33]. The earlier study [61] found a significant negative correlation between free plasma TRP and postpartum blues but did not perform a seasonal subgroup analysis due to its limited sample size.

The third study (*n* = 52) failed to find any significant association between free plasma TRP and postpartum blues despite using the same plasma assay and diagnostic method as the above two studies [34].

#### Postpartum blues and TRP/CAA ratio

Two studies calculated the TRP/CAA ratio and described its relationship to postpartum blues on days 1 to 5 postpartum [62, 64]. One study (*n* = 50) found a significant negative association between TRP/CAA ratio and postpartum blues in this period [62], whereas the other (*n* = 26) found no significant relationship [64].

## Discussion

Three key findings were derived from this study. Firstly, the meta-analysis demonstrated that low total TRP levels were significantly associated with PPD. Secondly, the descriptive analysis revealed that the absence of the total TRP spike in the early postnatal period showed a trending association with the development of PPD. Thirdly, we failed to find a significant association between free plasma TRP and PPD.

Our results support the hypothesis that PPD is associated with low circulating total TRP levels. As noted above the most widely accepted mechanisms by which TRP disposition is altered during pregnancy is via increased metabolic demand for TRP, altered albumin and NEFA levels, and hormonal fluctuations. However, we also postulate that the pathogenesis of PPD may be related to an alternative pathway for metabolism of TRP. As mentioned, 90% of TRP that is metabolised to kynurenine is due to the hepatic enzyme TDO, with a smaller proportion metabolised extrahepatically via the enzyme IDO. The induction of IDO via proinflammatory cytokines [30] leads to altered metabolism of TRP and has been previously implicated in a range of neuropsychiatric disorders [40, 66]. Compared to TDO, in non-pregnant individuals, IDO only results in modest quantities of TRP degradation to kynurenine. In proinflammatory states such as pregnancy, this contribution is greatly accentuated [30]. Indeed, one study found brain tryptophan availability (measured as TRP/CAA ratio) to be significantly and inversely related to several inflammatory markers [56]. More recently, a 2019 study found a significant association between IDO pleomorphism and depressive symptomatology postpartum [60]. However, a study by Abou-Saleh et al. [55] determined that the immunological activation of the kynurenine pathway immediately after delivery is unlikely. Given conflicting evidence, the role of IDO and inflammation as major factors in PPD remains unclear, and warrant further investigation [30, 67].

After parturition, TRP’s rate of return to normal can be divided into two stages. First, a rapid and supra-normal early rise on day 1–2 is observed, which may be explained by the sharp decline of NEFA in the immediate postpartum [34]. This initial peak is followed by a U-shaped dip and slower return to baseline levels over the following weeks [33, 34, 57]. Existing studies found that cases who failed to show the early TRP spike had significantly higher rates of depressive symptoms within the first five days of delivery [33, 57]. Additionally, the absence of the early TRP spike was predictive of major depression up to 6 months after childbirth [33]. The absence of the early TRP spike may be attributable to the rebound TDO activation following long-term suppression during pregnancy; however it is unclear why only certain individuals are affected, with an early study failing to find further associations with factors including age, duration of pregnancy and dietary compliance [33].

The third major finding of our meta-analysis is that there was no significant relationship between free plasma TRP and PPD. In the descriptive analysis, most papers found a negative correlation between free plasma TRP and PPD up to 10 days after childbirth, however this was not sustained in the meta-analysis where the difference was non-significant (SMD: -3.43, 95%CI [-7.76, 0.89]). Notably, one study found a significant negative correlation between free plasma TRP and EPDS, but was excluded from the meta-analysis because it did not provide sufficient quantitative data [55]. Given that a total of four studies reported on the relationship between free TRP and PPD, the exclusion of one major study from the meta-analysis may have impacted the results. Additionally, studies that examined late outcomes failed to find a significant relationship between free TRP and PPD at 2 months postpartum [59] and in a period up to 6 months postpartum [33].

The relevance of our findings for total TRP compared to free TRP are tentative. On one hand, an early rodent study showed that total plasma TRP was more predictive of brain TRP availability, measured as CSF concentration, than free TRP, and was therefore a better predictive tool for depression [68]. However, this finding contrasts with more extensive evidence that free TRP is in fact better correlated to brain TRP availability and a predictor for MDD [69, 70], which have been validated in human trials [71].

Studies examining the association between free plasma TRP and postpartum blues were highly heterogeneous in design, employing a variety of diagnostic scales. This heterogeneity is attributable to a current lack of consensus on the diagnostic criteria for postpartum blues, and hence the absence of a standardised and validated diagnostic tool. Consequently, existing studies produced inconsistent findings further confounded by seasonal variations in free TRP levels, implying that free plasma TRP is an unstable and unreliable marker of postpartum blues.

### Limitations

The findings of this meta-analysis were limited by the number of eligible studies identified, as well as the small sample sizes of many of the included studies. We determined to include the poorer quality studies due to the limited number of studies already available. Some of the studies failed to report whether the TRP levels measured were free or total. There was also a wide span in the years of publication (from 1980 through to 2019), which resulted in differences in the methodologies used to measure TRP levels. Further, due to the low number of eligible studies, we were unable to perform sub-group analyses and control for other confounders such as age, parity, marital status, socio-economic status, a history of depression, substance use and mode of delivery. Regarding the difference between our findings for total compared to free TRP levels, it is possible that some of the existing studies were not sufficiently powered to reveal a statistically significant correlation between free plasma TRP and PPD.

We recognise that total plasma levels of TRP may be imprecise and absolute plasma levels of TRP may have confounding factors related to dietary variation and pharmacological agents. Likewise, free TRP is considered a labile parameter influenced by many modulators, including fasting status, food intake, medications, exercise, stressors, and hormones [72]. Hence, blood samples are best taken under fasting conditions, as ingestion of food, lithium and antidepressants have been shown to affect plasma kynurenine and TRP levels [73]. Therefore, using a more controlled methodology for TRP collection in future studies is required to corroborate the results of this meta-analysis.

In addition, the most valid measure for the availability of TRP in the CNS is the TRP to competing amino acid (CAA) ratio ([TRP]/[CAA]). The use of free or total TRP alone as a marker for predicting overall TRP entry into the brain is only valid if the CAA levels remain stable. Unfortunately, very few studies reported on the quantitative association between [TRP]/[CAA] and PPD. However, it has been found that the decrease in the [TRP]/[CAA] ratio in MDD is mainly due to a decrease in TRP (rather than an increase in CAA levels) [44]. Hence, although [TRP]/[CAA} was seldom measured, it may still be reasonable to infer that low plasma TRP would be closely associated with low availability of TRP in the CNS.

Additionally, during the screening process, we noted that some authors utilised the kynurenine to TRP ratio, whilst other authors have used the brain tryptophan availability index. If further studies in this field were conducted, we propose measuring these as alternative biomarkers for PPD.

### Conclusion

To our knowledge, this meta-analysis is the first to quantitatively demonstrate that reduced total plasma TRP in the postpartum period is associated with PPD, and with postpartum depressive disorders. Our findings therefore support investigation of total serum TRP levels in the immediate postpartum period (within days 1–5) to determine its viability as a biomarker for the risk of developing PPD. Additionally, this may inform the use of supplementary post-partum dietary tryptophan in susceptible women.

Our findings support the hypothesis that postpartum blues is likewise associated with TRP depletion. To stratify the risk of developing PPD for women with postpartum blues, we propose further investigation regarding the relationship between low total TRP in women with postpartum blues and the subsequent development of PPD.

Our inability to perform a correlation meta-analysis to compare the relationship between TRP and depression scores highlights the necessity for greater consensus between study methodologies with regards to depression scales to enable this type of meta-analysis to occur.

## Supplementary Information

**Additional file 1.**

## Data Availability

All data analysed during this study are included in the published articles [and its supplementary information files]; or are available from the corresponding author on reasonable request.

## Abbreviations

BDI: Beck Depression Inventory
CAA: Competing amino acids
EPDS: Edinburgh Postnatal Depression score
IDO: Indoleamine 2,3-dioxygenase
KYN: Kynurenine
MAACL: Multiple Affect Adjective Checklist
MDD: Major depressive disorder
NEFA: Non-esterified fatty acids
PPD: Postpartum depression
SIGH-SAD: Structured Interview Guide for the Hamilton Depression Rating Scale-Seasonal Affective Disorder
STAI: State-Trait Anxiety Inventory
TDO: Tryptophan 2,3-dioxygenase
TRP: Tryptophan
VAS: Visual Analogue Scale
ZDS: Zung Depression Score
