# Supplementary material for: The association between tryptophan levels and postpartum mood disorders: a systematic review and meta-analysis
Source: BMC Psychiatry. 2022 Aug 8;22:539. doi: 10.1186/s12888-022-04178-6 (PMC9361669; doi:10.1186/s12888-022-04178-6)
Supplement: Supplementary file 1 — Additional file 1. [file 12888_2022_4178_MOESM1_ESM.docx]

**Supplementary Table 1.** Literature search strategy through OVID databases:

OVID MEDLINE:

1. Tryptophan/
2. Tryptophan.mp.
3. Kynurenine/
4. Kynurenine.mp.
5. 1 or 2 or 3 or 4
6. Exp Depression, Postpartum/
7. Postpartum depress*.mp. [mp=title, abstract, original title, name of substance word, subject heading word, floating sub-heading word, keyword heading word, organism supplementary concept word, protocol supplementary concept word, rare disease supplementary concept word, unique identifier, synonyms]
8. Postpartum period/ and exp Depression/
9. Postpartum blue*.mp.
10. Postpartum psych*.mp. [mp=title, abstract, original title, name of substance word, subject heading word, floating sub-heading word, keyword heading word, organism supplementary concept word, protocol supplementary concept word, rare disease supplementary concept word, unique identifier, synonyms]
11. 6 or 7 or 8 or 9 or 10
12. 5 and 11

EMBASE:

1. Tryptophan/
2. Tryptophan.mp.
3. Kynurenine/
4. Kynurenine.mp.
5. 1 or 2 or 3 or 4
6. Postnatal depression/
7. Postpartum depress*.mp.
8. Postpartum blue*.mp.
9. Postpartum psych*.mp.
10. 6 or 7 or 8 or 9 or 10
11. 5 and 11

PsycINFO:

1. Tryptophan/
2. Tryptophan.mp.
3. Kynurenine.mp.
4. 1 or 2 or 3
5. Exp Postpartum depression/
6. Postpartum depress*.mp.
7. Postnatal period/ and (“depression (emotion)”/ or exp Affective Disorders/)
8. Postpartum blue*.mp.
9. Postpartum psych*.mp.
10. 5 or 6 or 7 or 8 or 9
11. 4 and 10

Cochrane library:

1. Tryptophan/
2. Tryptophan.mp.
3. Kynurenine/
4. Kynurenine.mp.
5. 1 or 2 or 3 or 4
6. Exp Depression, Postpartum/
7. Postpartum depress*.mp.
8. Postpartum blue*.mp.
9. Postpartum psych*.mp.
10. 6 or 7 or 8 or 9
11. 5 and 10

**Supplementary Table 2.** List of excluded studies and reasoning

| **Reference** | **Reason** |
| --- | --- |
| 1. Achtyes E, Keaton SA, Smart L, Burmeister AR, Heilman PL, Krzyzanowski S, Nagalla M, Guillemin GJ, Galvis ML, Lim CK, Muzik M. Inflammation and kynurenine pathway dysregulation in post-partum women with severe and suicidal depression. Brain, behavior, and immunity. 2020 Jan 1;83:239-47. | Depression onset not exclusive to postpartum period |
| 1. Nazzari S, Molteni M, Valtorta F, Comai S, Frigerio A. Prenatal IL-6 levels and activation of the tryptophan to kynurenine pathway are associated with depressive but not anxiety symptoms across the perinatal and the post-partum period in a low-risk sample. Brain, Behavior, and Immunity. 2020 Oct 1;89:175-83. | Depression onset not exclusive to postpartum period |
| 1. Duan KM, Wang SY, Yin JY, Li X, Ma JH, Huang ZD, Zhou YY, Yu HY, Yang M, Zhou HH, Liu ZQ. The IDO genetic polymorphisms and postpartum depressive symptoms: An association study in Chinese parturients who underwent cesarean section. Archives of women's mental health. 2019 Jun;22(3):339-48. | No data for outcome of interest |
| 1. Van Lee L, Cai S, Loy SL, Tham EK, Yap FK, Godfrey KM, Gluckman PD, Shek LP, Teoh OH, Goh DY, Tan KH. Relation of plasma tryptophan concentrations during pregnancy to maternal sleep and mental well-being: The GUSTO cohort. Journal of affective disorders. 2018 Jan 1;225:523-9. | No data for outcome of interest |
| 1. Keaton SA. *The Role of Inflammation and the Kynurenine Pathway in Mood Disorders and Pregnancy* (Doctoral dissertation, Michigan State University). | No data for outcome of interest |
| 1. Kurup RK, Kurup PA. Hypothalamic digoxin and hypomagnesemia in human pre‐eclampsic toxemia, cortical venous thrombosis, and postpartum psychosis. The Journal of Trace Elements in Experimental Medicine: The Official Publication of the International Society for Trace Element Research in Humans. 2002;15(4):171-90. | No data for outcome of interest |
| 1. Teshigawara T, Mouri A, Kubo H, Nakamura Y, Shiino T, Okada T, Morikawa M, Nabeshima T, Ozaki N, Yamamoto Y, Saito K. Changes in tryptophan metabolism during pregnancy and postpartum periods: Potential involvement in postpartum depressive symptoms. Journal of affective disorders. 2019 Aug 1;255:168-76. | No data for outcome of interest |
| 1. Sha Q, Achtyes E, Nagalla M, Keaton S, Smart L, Leach R, Brundin L. Associations between estrogen and progesterone, the kynurenine pathway, and inflammation in the post-partum. Journal of affective disorders. 2021 Feb 15;281:9-12. | Duplicated study cohort |
| 1. Maes M, Verkerk R, Bonaccorso S, Ombelet W, Bosmans E, Scharpé S. Depressive and anxiety symptoms in the early puerperium are related to increased degradation of tryptophan into kynurenine, a phenomenon which is related to immune activation. Life sciences. 2002 Sep 6;71(16):1837-48. | Duplicated study cohort |
